# Use of Second-Generation Basal Insulin Gla-300 in Special Populations: A Narrative Mini-Review

**DOI:** 10.2174/1573399819666230109113205

**Published:** 2023-08-02

**Authors:** Sujoy Ghosh, Sanjay Kalra, Ganapathi Bantwal, Rakesh Kumar Sahay

**Affiliations:** 1 Department of Endocrinology, IPGME & R, Kolkata, West Bengal, India;; 2 Department of Endocrinology, Bharti Hospital, Karnal, Haryana, India;; 3 Department of Endocrinology, St. John’s Medical College & Hospital, Bengaluru, Karnataka, India;; 4 Department of Endocrinology, Osmania Medical College, Hyderabad, Telangana, India

**Keywords:** Elderly, hypoglycemia, Ramadan, renal impairment, special population, T1DM, Gla-300, insulin, glargine

## Abstract

**Background and Aims:**

Hypoglycemia and insulin-related adverse events are crucial barriers to effective diabetes management, particularly in the elderly, people with renal impairment, people with diabetes fasting during Ramadan, or people with type 1 diabetes mellitus (T1DM). There is a scarcity of clinical and real-world evidence assessing the effectiveness and safety of insulin glargine 300 U/mL (Gla-300) in these special populations. To understand the entirety of evidence, this mini-review elaborates on the use of Gla-300 in diabetes management among special populations.

**Methods:**

Clinical and real-world evidence related to the use of Gla-300 among special populations with diabetes were retrieved using PUBMED and Google Scholar.

**Results:**

Gla-300 has shown improved glycemic control with stable insulin action and low risk of hypoglycemia in diverse groups with diabetes. It also appears to have an acceptable safety profile during Ramadan fasting. However, adequate monitoring and adjustment of insulin dose on an individual basis should be considered.

**Conclusion:**

Gla-300 is a second-generation basal insulin with proven benefits of reduced risk of hypoglycemia and improved glycemic control in special populations of people with diabetes.

## 1 INTRODUCTION

Basal insulin (BI) therapy is a widely used option among available insulin therapies and requires a balance between achieving individualized glycemic targets and reducing hypoglycemia. Multiple risk factors attributable to the increased risk of hypoglycemia are older age, renal impairment, long duration of diabetes, history of severe hyperglycemia, and the presence of comorbidities [1-6]. However, an increased risk of hypoglycemia and associated fear of first-generation BIs are the obstructing factors in their continued use among these vulnerable populations. Second-generation BI analogues, insulin glargine 300 U/mL (Gla-300) and insulin degludec (IDeg), have been increasingly used for diabetes management among clinicians as they provide a wide range of advantages including longer duration and stable insulin action, and comparable glycemic efficacy with lower hypoglycemia risk compared to the first-generation BI analogues [7-10]. Growing evidence suggests that these second- generation BI analogues have potential benefits if used in special populations with diabetes at high risk of hypoglycemia. This mini-review describes the role of Gla-300 in achieving improved glycemic control and low risk of hypoglycemia among special populations with diabetes, including older people, people with renal impairment, type 1 diabetes mellitus (T1DM), and people fasting during Ramadan.

## 2 METHODOLOGY

Clinical and real-world evidence related to the use of Gla-300 among special populations with diabetes (including older age groups, people with renal impairment, people with T1DM, and people fasting during Ramadan) and published up to 06 October 2021 were searched using PUBMED and Google Scholar. The following search terms were used in combination with appropriate MeSH terms: Chronic kidney disease, CKD, elderly, insulin, Glargine, U-300, Gla-300, hypoalbuminemia, Ramadan, renal impairment, type 1 diabetes mellitus, T1DM, type 2 diabetes, and T2DM. A manual search was further performed through internet and the references of the identified articles were scanned to find the studies that were not retrieved through the electronic database search; these included published congress abstracts as well.

## 3 GLA-300 IN SPECIAL POPULATION

Table **1** summarizes key observations of important studies discussed in each of the following sections.

### 3.1 Renal Impairment

Renal impairment is an independent risk factor for hypoglycemia which increases the risk of hypoglycemia in people with type 2 diabetes mellitus (T2DM) [11, 12]. A strong evidence base indicates that diabetes management in this population is complex and increased hypoglycemia risk associated with cardiovascular morbidity and mortality, ultimately limits the options for glucose-lowering therapy [13-16]. Owing to the high risk of hypoglycemia and reduced renal clearance in people with T2DM and chronic kidney disease (CKD), an insulin treatment that decreases glycated hemoglobin (HbA1c) without escalating the risk of hypoglycemia is clinically important. Subgroup analyses according to renal function categorization from various randomized controlled trials (RCTs) and real-world studies have provided promising results with Gla-300 use. A meta-analysis of pooled 6-month data from EDITION 1, 2, and 3 trials according to renal function subgroups shows comparable glycemic control (decrease in HbA1c and the proportion of people achieving HbA1c targets) with Gla-300 *vs.* glargine 100 U/mL (Gla-100) at once-daily dosage in T2DM cohort. Furthermore, Gla-300 offered a lower risk of nocturnal confirmed or severe hypoglycemia (*vs*. Gla-100), irrespective of renal function subgroups. These findings indicate the benefit of reduced risk of hypoglycemia with Gla-300 in individuals with T2DM and impaired renal function [17]. In addition, the BRIGHT study, which compared two second-generation BIs (Gla-300 *vs.* IDeg-100) head-to-head, has provided important information about the effectiveness and safety of Gla-300 in insulin-naïve individuals with T2DM and renal impairment. The heterogeneity of treatment effect across renal function subgroups (*P*=0.02) and greater reduction in HbA1c with Gla-300 *vs.* IDeg-100 among those with estimated glomerular filtration rate (eGFR) <60 mL/min/1.73 m^2^ (mean difference: -0.43% (95% CI: -0.74%, -0.12%)) were observed. Hypoglycemia incidence over the 24 weeks was similar across both the treatment arms; thus, suggesting better effectiveness of Gla-300 over IDeg-100 without compromising safety [18]. A recent report from REALI CKD pooled analysis also demonstrated concordant observations in terms of the clinically significant glycemic control with favorable hypoglycemia risk in a diverse group of renal function subgroups (baseline eGFR: ≥90, 60-89, 45-59, and 15-44 mL/min/1.73 m^2^) indicating no influence of baseline renal function on the effectiveness and safety of Gla-300 during six months of treatment in individuals with poorly controlled T2DM [19]. Further additional studies in individuals with more advanced renal impairment (eGFR <45 or <30 mL/min/1.73 m^2^) would be useful. Coming to the real-world evidence, currently, four studies are available that provide corroborating observations to the above-mentioned trials. In DELIVER D+ study, analysis of people with moderate-to-severe renal impairment demonstrated the effectiveness of Gla-300 in achieving improved glycemic control and lower hypoglycemia incidence after switching from a first-generation BI analogue (Gla-100/detemir) [20]. On the contrary, observations from DELIVER High Risk study showed that switching to Gla-300 resulted in significantly lower risk of hypoglycemia (*P*=0.006) and emergency department/hospitalization related to hypoglycemia (*P*=0.022) in people with moderate-to-severe renal impairment [21]. The LIGHTNING study demonstrated significantly lower predicted rates of severe hypoglycemia with Gla-300 use (*vs*. first-generation BI) [22]. In accordance with the previously discussed evidence, the ACHIEVE Control study also showcased the potential benefit of Gla-300 (*vs*. standard-of-care BI analogue) in terms of hypoglycemia avoidance in those with renal impairment; particularly in those with moderate-to-severe renal impairment for a 1-year treatment duration (odds ratio >1.40) [23]. Another real-world study, ATOS (NCT03703869), was conducted prospectively across multiple countries (n = 388) for a period of one-year (n = 4589 participants) to assess the clinical effectiveness, safety and health-economic benefits of Gla-300 initiation in insulin-naïve people with T2DM who failed to achieve glycemic control with OADs [24]. A subgroup analysis of ATOS study also indicated the effectiveness of Gla-300 in improving glycemic control and reducing hypoglycemia risk independent of an individual’s renal impairment history [25]. In summary, the aforementioned real-world studies provide complementary findings that support results from RCTs and reflect the effectiveness and safety of Gla-300 from routine clinical practice in people with T2DM and renal impairment.

### 3.2 Hypoalbuminemia

The only study evaluating the differential effect of hypoalbuminemia on hypoglycemia in 30 individuals with T2DM treated with Gla-300 and IDeg reported that in those with serum-albumin levels <3.8 g/dL, the mean percentages of time with hypoglycemia (1.9 *vs.* 11.1; *P*=0.002), clinically important hypoglycemia (0.02 *vs.* 3.64, *P*=0.002), and nocturnal hypoglycemia (1.5 *vs.* 7.4; *P*=0.004) were lower in Gla-300 *vs.* IDeg group [26]. Therefore, it may be necessary to consider the risk of nocturnal hypoglycemia based on albumin levels when using IDeg; however, the use of Gla-300 may be unaffected by albumin levels. This is an interesting finding showing a signal of benefit for Gla-300 use. It should be noted that this study had a small sample size (n = 30); therefore, more evidence and large-scale studies are needed to corroborate these findings.

### 3.3 Older Population

Older people with diabetes are more prone to hypoglycemia and impaired awareness of hypoglycemia than younger populations [27, 28]. Older individuals with the incidence of hypoglycemia during the initial phase of BI treatment are at high risk of hospitalization or death [29]. Therefore, minimizing the hypoglycemia risk is vital and several clinical and real-world studies have evaluated efficacy/effectiveness and safety of Gla-300 in older people with diabetes.

The SENIOR trial demonstrated that the Gla-300 was effective in older people (≥65 years) with lower rates of documented symptomatic hypoglycemia at any time of day (24 hours) *vs.* Gla-100, particularly in those aged ≥75 years (rate ratio 0.45 [95% CI, 0.25, 0.83]) [30]. In the pooled analysis of EDITION trials, at the end of six months, compared to Gla-100, Gla-300 demonstrated a reduced risk of nocturnal confirmed or severe hypoglycemia and documented symptomatic hypoglycemia along with a comparable glycemic control in older (≥65 years) population with T2DM. Additionally, Gla-300 provided the benefit of achieving glycemic control without hypoglycemia over Gla-100 [31].

Furthermore, a pooled analysis of EDITION 2 and 3 trials reported an additional benefit of less weight gain associated with Gla-300 *vs.* Gla-100 among older (>55 years) people with T2DM [32]. On the contrary, BRIGHT Elderly study (post hoc analysis of BRIGHT) reported apparently greater HbA1c reduction and similar hypoglycemia risk with Gla-300 *vs.* IDeg in older adults (≥70 years). In the initial 12-week titration period, Gla-300 group showed consistently lower incidence and rates of anytime and nocturnal severe or confirmed hypoglycemia compared with IDeg group irrespective of age subgroups (</≥65 years and </≥70 years) [33, 34].

Real-world evidence from DELIVER 3 study involving older individuals (≥65 years) with T2DM mirrored observations from clinical trials. Switching to Gla-300 *vs.* first-generation BIs was associated with greater/similar improvements in glycemic control and Gla-300 significantly reduced the risk of hypoglycemia in this population [35]. Similarly, the observations of the REALI pooled analysis indicate the potential use of Gla-300 in older people prone to hypoglycemia. The incidences of symptomatic hypoglycemia during any time of the day (5.9% *vs.* 7.6%-9.4% for the younger subgroups) or night-time (0.5% *vs.* 1.6%-2.5%) were lower in those aged ≥80 years compared to younger age subgroups (subgroups:<50, 50-59, 60-69, 70-79 years) [36]. With similar hypoglycemia risk, evidence from X-STAR study reassures safety of Gla-300 in T2DM cohort of older age or with renal impairment [37]. A subgroup analysis of ATOS study indicated that Gla-300 was beneficial in attaining target HbA1c and reduced risk of hypoglycemia among people with T2DM irrespective of their age groups [38].

To summarize, all of these studies show that Gla-300 has a low risk of hypoglycemia across a wide age range (>55 to 80 years) while maintaining glycemic control.

### 3.4 People with T1DM

Extensive literature search has identified multiple studies evaluating pharmacokinetic-pharmacodynamic (PK-PD) profile of Gla-300 in comparison to other BIs in people with T1DM. These data demonstrate less glycemic variability and stable glucose profile when administered at any time of the day indicating possible flexibility of dosing [7, 8, 39, 40]. Furthermore, the individualized, clinical doses of Gla-300 and Gla-100 resulted in a similar euglycemic potential under steady state conditions; however, Gla-300 administered in the evening exhibited a more stable profile, with lower variability and more physiological modulation of hepatic glucose production compared with Gla-100 [41]. Therefore, Gla-300 (if administered in the evening) can be advantageous in terms of lowering the risk of nocturnal hypoglycemia, improving pre-dinner hyperglycemia, and reducing within-day glucose variability in those with T1DM. Gla-300 showed 20% lesser within-day glycemic variability when compared with IDeg-100 in a euglycemic clamp study in individuals with T1DM [39]. On the contrary, Heise *et al*. showed significantly lower relative within-day and day-to-day variability with IDeg compared to Gla-300 [42]. However, yet another study conducted by Lucidi *et al*. demonstrated that Gla-300 had lower within-day variability compared to IDeg-100 despite even distribution of insulin activity over 24 h post-dosing [40].

Comparison of Gla-300 with Gla-100 in EDITION 4 trial reported comparable HbA1c reduction with similar incidence of overall hypoglycemia. However, Gla-300 showed reduced nocturnal hypoglycemia incidence during the initial titration period in T1DM [43]. Furthermore, several other RCTs have established supporting evidence for Gla-300 use over other BIs in terms of lower risk of nocturnal or severe hypoglycemia [44-46]. A pooled analysis of EDITION 4, EDITION JP1, and EDITION JUNIOR trials also revealed lower risk of severe hypoglycemia with Gla-300 *vs.* Gla-100 [43]. Switching to Gla-300 once-daily from other BI (twice-daily) analogues improved glycemic control and treatment satisfaction among people with T1DM [47, 48]. These observations are replicated in real-world clinical practice as well [49, 50].

Further, the real-world evidence from across the world supports the above-discussed benefits of Gla-300 in T1DM management along with the highest persistence rate [50-52]. In particular, Gla-300 has been shown to be effective in substantial improvement in HbA1c reduction with reduced incidence of nocturnal or severe hypoglycemia episodes and diabetic ketoacidosis [53, 54]. The REALITY study from Canada demonstrated that those with T1DM switching to Gla-300 had significant reductions in HbA1c with no change in weight or insulin dose [55]. Similarly, the recent RESTORE-1 study showed that switching from first-generation BI to Gla-300/IDeg was associated with similar improvements in glycemic control and overall significant decrease in hypoglycemia, with no severe events in Gla-300 group *vs.* seven events in IDeg group (*P=*0.02) [56].

Further, the OneCARE real-world study has demonstrated a comparable safety and effectiveness profile of Gla-300 and IDeg in terms of achieving a full day time in range (TIR) 70-180 mg/dL; however, those receiving Gla-300 spent significantly higher time in target glucose range (TIR 70-140 and 70-180 mg/dL) and lower time above range (>180 mg/dL) at night-time than IDeg, indicating the lower risk of nocturnal hypoglycemia with Gla-300 use [57].

A post-marketing surveillance data from Japanese X-STAR study highlights the safety of Gla-300 with no unprecedented adverse events observed during 1-year of study period. Due to blunted up-titration (+1.13 U/day [+0.02 U/kg/day]) of the Gla-300 dose, the HbA1c reduction over 12 months (-0.12%) was slightly moderate in this real-world study compared with the clinical trial data [58-60]. Therefore, for better glycemic control, clinicians should apply vigilant up-titration of Gla-300 with a careful consideration for hypoglycemia risk in a real-world setting. To summarize, the overall evidence suggests the potential benefit of Gla-300 among the T1DM population in terms of lower risk of nocturnal or severe hypoglycemia, improved glycemic control, and better treatment satisfaction compared to first-generation BIs.

### 3.5 Ramadan

Management of diabetes during Ramadan fast is a crucial challenge for insulin users as long hours of fasting can make them more susceptible to potential complications (severe hypoglycemia, hyperglycemia, and ketoacidosis). Real-world evidence from ORION study showed overall low incidence of severe and/or symptomatic hypoglycemia, with no severe hypoglycemic events reported during Ramadan and clinically improved glycemic control from pre-Ramadan to post-Ramadan period [61]. Therefore, Gla-300 seems to be a promising insulin therapy for diabetes management in people fasting during the Ramadan period.

## CONCLUSION

Gla-300 is a second-generation BI with proven benefits of reduced risk of hypoglycemia and improved glycemic control in special population of people with diabetes, including those with renal impairment, older individuals, and T1DM. It also appears to have an acceptable safety profile during Ramadan fasting. However, while using Gla-300 in these special populations, adequate monitoring and adjustment of insulin dose on an individual basis should be considered. Future clinical and real-world studies exploring use of Gla-300 will provide more information on its effectiveness and safety.

## DISCLOSURES

All authors had full access to the articles reviewed in this manuscript, have read and reviewed the final draft of this manuscript and take complete responsibility for the integrity and accuracy of this manuscript. The content published herein solely represents the views and opinions of the authors. The details published herein are intended for informational, educational, academic and/or research purposes and are not intended to substitute for professional medical advice, diagnosis or treatment.

## Figures and Tables

**Table 1:** Summary of evidence.

**Study Details**	**Study Cohort (Sample Size)**	**Treatment**	**Summary of Key Observations**
**Renal Impairment**			
A meta-analysis of pooled 6-month data from EDITION 1, 2, and 3 [17]	T2DM with mild-to-moderate renal impairment (n=2496)	Gla-300 *vs.* Gla-100	• Comparable glycemic control (decrease in HbA1c and the proportion of people achieving HbA1c targets) with Gla-300 *vs.* Gla-100 at once-daily dosage. • Gla-300 offered a lower risk of nocturnal confirmed or severe hypoglycemia (*vs*. Gla-100) irrespective of renal function subgroups (eGFR <60 mL/min/1.73 m^2^: RR 0.76 [95% CI 0.62-0.94] and eGFR ≥60 mL/min/1.73 m^2^: RR 0.75 [95% CI 0.67-0.85]).
BRIGHT trial, a head-to-head trial comparing two second-generation BIs [18]	Insulin-naïve people with T2DM and renal impairment Gla-300: n=465 IDeg-100: n=463	Gla-300 *vs.* IDeg-100	• The heterogeneity of treatment effect across renal function subgroups (*P*=0.02) and greater reduction in HbA1c with Gla-300 *vs.* IDeg-100 among those with eGFR <60 mL/min/1.73 m^2^ (mean difference: -0.43% [95% CI: -0.74%, -0.12%]). • Hypoglycemia incidence over the 24 weeks was similar.
REALI CKD pooled analysis [19]	T2DM with poor glycemic control (n=1712) baseline eGFR: ≥ 90 (n=599), 60-89 (n=786), 45-59 (n=219), and 15-44 mL/min/1.73 m^2^ (n=108)	Gla-300	• Clinically significant glycemic control with favorable hypoglycemia risk in a diverse group of renal function subgroups indicating no influence of baseline renal function on the effectiveness and safety of Gla-300 during six months of treatment.
DELIVER D + study [20]	T2DM with moderate-to-severe renal impairment Gla-300 switchers: n [HbA1c reductions/hypoglycemia] =232/460	Gla-300 switchers from Gla-100/IDet	• Gla-300 resulted in improved glycemic control (0.58%) and lower hypoglycemia incidence (18.5%) after switching from a first-generation BI analogue (Gla-100/IDet).
DELIVER High Risk [21]	T2DM with moderate-to-severe renal impairment (n=861)	Gla-300 *vs.* Gla-100/IDet	• Switching to Gla-300 resulted in significantly lower risk of hypoglycemia (*P*=0.006) and emergency department/hospitalization related to hypoglycemia (*P*=0.022).
LIGHTNING study [22]	T2DM with moderate-to-severe renal impairment (BI switcher: n=15,889) (Insulin naïve: n=19,205)	Gla-300 *vs.* first-generation BI (IDet/Gla-100)	• Significantly lower predicted rates of severe hypoglycemia with Gla-300 use *vs.* IDet (0.31% *vs.* 0.42%; *P <* 0.05) in BI switcher cohorts and Gla-300 *vs.* Gla-100/IDet in insulin naïve subgroups (0.10% *vs.* 0.19%; *P <* 0.05).
ACHIEVE Control study [23]	Insulin naïve people with T2DM with moderate-to-severe renal impairment (n=3304)	Gla-300 *vs.* Gla-100/ IDet	• Potential benefit of Gla-300 (*vs*. standard-of-care BI analogue) in terms of hypoglycemia avoidance for a 1-year treatment duration (odds ratio >1.40).
ATOS study- subgroup analysis [25]	Insulin naïve people with T2DM along with (n=581) or without (n=3841) a history of renal impairment	Gla-300	In people with renal impairment *vs.* without renal impairment: • Improved glycemic control (HbA1c reductions from baseline to Month 6 [-1.50 *vs.* -1.50%] and 12 [-1.84 *vs.* -1.88%]). • Reduced hypoglycemia risk (0.66 *vs.* 0.021 event rate per person-year at Month 6 and 0.050 *vs.* 0.017 event rate per person-year at Month 12) independent of persons’ renal impairment history.
**Hypoalbuminemia**			
Evaluation of the differential effect of hypoalbuminemia on hypoglycemia [26]	T2DM with serum-albumin levels <3.8 g/dL [n=30]	Gla-300 *vs.* IDeg	• Mean percentages of time with hypoglycemia (1.9 *vs.* 11.1; *P*=0.002), clinically important hypoglycemia (0.02 *vs.* 3.64, *P*=0.002), and nocturnal hypoglycemia (1.5 *vs.* 7.4; *P*=0.004) were lower in Gla-300 *vs.* IDeg group.
**Older Population**			
SENIOR trial [30]	Older (≥65 years) population with T2DM (n=1014)	Gla-300 *vs.* Gla-100	• Gla-300 was effective in older people (≥65 years) with lower rates of documented symptomatic hypoglycemia at any time of day (24 hour) *vs.* Gla-100 (Gla-300: 1.85; Gla-100: 2.56; rate ratio 0.74 [95% CI, 0.56, 0.96], particularly in those aged ≥75 years (Gla-300: 1.12; Gla-100: 2.71; rate ratio 0.45 [95% CI, 0.25, 0.83]).
Pooled analysis of EDITION trials [31]	Older (≥65 years) population with T2DM (n= 662)	Gla-300 *vs.* Gla-100	• At the end of six months, Gla-300 demonstrated reduced risk of nocturnal confirmed or severe hypoglycemia and documented symptomatic hypoglycemia (relative risk: 0.70 [0.57 to 0.85]) along with a comparable glycemic control. • Gla-300 provided a benefit of achieving glycemic control without hypoglycemia over Gla-100.
Pooled analysis of EDITION 2 and 3 trials [32]	Older (>55 years) population with T2DM (n=1117)	Gla-300 *vs.* Gla-100	• Less weight gain associated with Gla-300 *vs.* Gla-100 at 6 months (P=0.027) and at 12 months (*P*=0.021).
BRIGHT Elderly study (post hoc analysis of BRIGHT) [33, 34]	Older (≥70 years) population with T2DM (n=161)	Gla-300 *vs.* IDeg-100	• Greater HbA1c reduction (-1.69% *vs.* -1.34%) and similar hypoglycemia risk with Gla-300 *vs.* IDeg-100.
DELIVER 3 study [35]	Older (≥65 years) population with T2DM (n=1176)	Gla-300 *vs.* first-generation BIs (Gla-100/IDet)	• Switching to Gla-300 *vs.* first-generation BIs was associated with greater/similar improvements in glycemic control. • Gla-300 significantly reduced the risk of hypoglycemia (event rate: adjusted rate ratio: 0.63, 95% CI: 0.53-0.75; *P <* 0.001).
REALI pooled analysis [36]	T2DM cohort <50 years (n=727), 50–59 years (n=2030), 60–69 years (n=3054), 70–79 years (n=1847), ≥80 years (n=448)	Gla-300	• Incidences of symptomatic hypoglycemia during any time of the day (5.9% *vs.* 7.6%-9.4%) or night-time (0.5% *vs.* 1.6%-2.5%) were lower in people aged ≥80 years compared to younger age subgroups, respectively (subgroups:<50, 50-59, 60-69, 70-79 years).
X-STAR study [37]	T2DM cohort of older age (n=4621)	Gla-300	• Similar hypoglycemia risk (2.9–3.5% and 2.7–5.2% of insulin naïve and insulin-experienced people, respectively, for age subgroups [<65, 65, ≤75, and ≥75 years]).
ATOS study- subgroup analysis [38]	T2DM older adults <70 years (n=3908) ≥70 years (n=514)	Gla-300	• Gla-300 was beneficial in attaining target HbA1c and reduced risk of hypoglycemia among people with T2DM irrespective of their age groups.
**Type 1 Diabetes Mellitus (T1DM)**			
EDITION 4 trial [43]	T1DM (n=549)	Gla-300 *vs.* Gla-100	• Comparable HbA1c reduction (difference, 0.04% [95% CI -0.10 to 0.^19^]). • Similar incidence of overall hypoglycemia. • Gla-300 showed reduced nocturnal hypoglycemia incidence during the initial titration period (rate ratio 0.69 [95% CI 0.53 to 0.91].
A meta-analysis of 6-month phase 3 clinical trials (EDITION 4, EDITION JP 1 and EDITION JUNIOR) [44]	T1DM Gla-300 (n=629) Gla-100 (n=626)	Gla-300 *vs.* Gla-100	• Lower risk of severe hypoglycemia with Gla-300 (Hazard ratio 0.65 [95% CI 0.44 to 0.98; *P*=0.0^38^]). • ≥1 severe hypoglycemia event with Gla-300 (6.2%) than with Gla-100 (9.3%).
EDITION JUNIOR trial [45]	Children and Adolescents (6–17 years) with T1DM (n=463)	Gla-300 *vs.* Gla-100	• Comparable glycemic reduction from baseline to week 26 (LS mean [SE] -0.40% [0.06%] for both treatment arms with LS mean difference 0.004% [95% CI -0.17 to 0.^18^]). • Similar incidence and event rates of severe or documented hypoglycemia (6.0% with Gla-300 and 8.8% with Gla-100; relative risk 0.68 [95% CI 0.35 to 1.^30^]).
EDITION JP 1 study [46]	Adults with T1DM (n=243)	Gla-300 *vs.* Gla-100	• Gla-300 was non-inferior to Gla-100 for HbA1c change over the 6-month period (LS mean difference 0.13% [95% CI −0.03 to 0.29]). • The annualized rate of confirmed or severe hypoglycemic events was 34% lower with Gla-300 than with Gla-100 at night (rate ratio 0.66 [95% CI 0.48 to 0.92]) and 20% lower at any time of day (24 h; rate ratio 0.80 [95% CI 0.65 to 0.9]).
Phase 4 OPTIMIZE Study [47]	Adults with T1DM on basal-bolus insulin (n=94)	Switch from twice-Daily BI (IDet/ Gla-100/ NPH insulin) to Once-Daily Gla-300	• Improved glycemic control (mean HbA1c 8.54% at baseline and 8.27% at week 24; mean difference 0.27% [95% CI 0.15 to 0.^40^]; *P <* 0.0001). • Significant improvements in treatment satisfaction were observed (DTSQs total scores from baseline [24.^1^] to week 24 [29.^4^]; *P <* 0.0001).
Phase 4 TOP1 trial (Brazilian cohort) [48]	T1DM (n=123)	Switch from twice-Daily BI to Once-Daily Gla-300 in combination with a prandial rapid-acting insulin analog	• A significant reduction from run-in to the last 4 weeks on treatment in the proportion of people with at least one hypoglycemic event (*P*=0.025), as well as decreased numbers of hypoglycemic events/person-year of any type, symptomatic, and confirmed (*P*=0.036, 0.007, and 0.049, respectively). • A significant improvement in individuals’ satisfaction (Diabetes Treatment Satisfaction Questionnaire Total: 24.8 *vs.* 30.7; *P <* 0.001) and perceived hyperglycemia (4.0 *vs.* 3.0; *P <* 0.001) scores.
Toujeo-Neo study [49]	Adults with T1DM (n=397)	Switching to Gla-300 from basal-bolus insulin	• HbA1c reduction by 0.48% with a trend of lower nocturnal hypoglycemia.
A descriptive real-life study [50]	Adults with T1DM (n=247)	Switching to Gla-300 from Gla-100	• Proportion of individuals with HbA1c values <7.5% increased at 6 months (33.5 *vs.* 40.5%; *P <* 0.05) and remained stable during one year of follow-up. • Hypoglycemic events significantly decreased after one year of treatment in people with history of hypoglycemia.
A Canadian Retrospective Cohort Study [51]	T1DM and T2DM combined (n not available)	Gla-300, Gal-100, IDet	• 63% of people taking Gla-300 persisted with therapy compared to 54% and 50% of Gla-100, 45% of IDet, and 29% of NPH individuals.
Analysis of DPV and DIVE registries [52]	T1DM and T2DM (n=7268)	Gla-300 *vs.* Gla-100	• Severe hypoglycemia was less prevalent in T2DM and among recipients of Gla-300 (3.1 *vs.* 3.9%), whereas in T1DM the rate was higher (10.6 *vs.* 10.1%) compared to Gla-100.
SPARTA [53]	Adults with T1DM (n=298)	Gla-300	• Mean reduction in HbA1c from baseline to month 6 was –4 mmol/mol (-0.4%; *P <* 0.001). • Severe hypoglycemic episodes were documented for 6/298 and 4/298 participants and diabetic ketoacidosis episodes requiring emergency department visits or hospitalization were documented for 4/298 and 6/298 participants, before and after Gla-300 initiation.
A retrospective observational study from Belgium [54]	Adults with T1DM (n=116)	Switching to Gla-300 from Gla-100	• At the end of 24 weeks after switching, HbA1c decreased from 8.0% to 7.9% (*P*=0.03). • Nocturnal hypoglycemic rate declined quickly after the switch (22.2% *vs.* 12.2%; relative risk 0.46 [95% CI 0.30 to 0.68]; *P <* 0.0001).
REALITY study [55]	Adults with T1DM and T2DM T1DM (n=187) T2DM (n=396)	Switching to Gla-300 from Gla-100	• Significant reductions in HbA1c (T2DM: -0.45%; *P <* 0.001 and T1DM: -0.17%; *P*=0.04) with no change in weight or insulin dose.
RESTORE-1 Study [56]	Adults with T1DM (n=585)	Switching to Gla-300/IDeg-100 from first-generation BI	• Similar improvements in glycemic control (-0.20%; [95% CI -0.32; -0.0^8^] in the Gla-300 group and -0.14%; [95% CI -0.24; -0.04) in the IDeg-100 group). • Overall significant decrease in hypoglycemia (Incidence rate ratios 0.82 [95% CI 0.55; 1.^22^] for Gla-300 and 0.83; [95% CI 0.38; 1.83] for IDeg-100), with no severe events in Gla-300 group *vs.* seven events in IDeg group (*P*=0.02).
OneCARE Study [57]	Adults with T1DM (n=199)	Gla-300 *vs.* IDeg 100	• People receiving Gla-300 spent significantly higher time in target glucose range (TIR 70-140 mg/dL: 31.8% *vs.* 26.9%; *P*=0.0182 and TIR 70-180 mg/dL: 52.4% *vs.* 46.2%, *P*=0.0209) and lower time above range (>180 mg/dL) at night-time (40.1% *vs.* 47.2%, *P*=0.0199) than IDeg.
X-STAR study [59]	Adults with T1DM (n=774)	Insulin-experienced individuals initiated on Gla-300	• No unprecedented adverse events observed during 1-year of study period indicate safety of Gla-300.
**Ramadan**			
ORION study [61]	Adults with T2DM who fast during Ramadan (n=502)	Gla-300	• Clinically improved glycemic control from pre-Ramadan (8.1%) to post-Ramadan period (7.6%) (mean HbA1c reduction= -0.4%). • Overall low incidence of severe and/or symptomatic hypoglycemia (2.6%), with no severe hypoglycemic events reported during Ramadan.

**Abbreviations:** BI, basal insulin; CI, confidence interval; CKD, chronic kidney disease; eGFR, estimated glomerular filtration rate; Gla-100, insulin glargine 100 U/mL; Gla-300, insulin glargine 300 U/mL; HbA1c, glycated hemoglobin; IDeg-100, insulin degludec 100 U/mL; IDet, insulin detemir; RR; relative risk; T1DM, type 1 diabetes mellitus; T2DM, type 2 diabetes mellitus.

## References

[1] Zoungas S., Patel A., Chalmers J. (2010). Severe hypoglycemia and risks of vascular events and death.. N. Engl. J. Med..

[2] Yun J.S., Ko S.H. (2016). Risk factors and adverse outcomes of severe hypoglycemia in type 2 diabetes mellitus.. Diabetes Metab. J..

[3] Wysham C., Bhargava A., Chaykin L. (2017). Effect of insulin degludec vs. insulin glargine U100 on hypoglycemia in patients with type 2 diabetes: the SWITCH 2 randomized clinical trial.. JAMA.

[4] Huang E.S., Laiteerapong N., Liu J.Y., John P.M., Moffet H.H., Karter A.J. (2014). Rates of complications and mortality in older patients with diabetes mellitus: the diabetes and aging study.. JAMA Intern. Med..

[5] Davis T.M.E., Brown S.G.A., Jacobs I.G., Bulsara M., Bruce D.G., Davis W.A. (2010). Determinants of severe hypoglycemia complicating type 2 diabetes: the Fremantle diabetes study.. J. Clin. Endocrinol. Metab..

[6] Amiel S.A., Dixon T., Mann R., Jameson K. (2008). Hypoglycaemia in type 2 diabetes.. Diabet. Med..

[7] Becker R.H.A., Nowotny I., Teichert L., Bergmann K., Kapitza C. (2015). Low within- and between-day variability in exposure to new insulin glargine 300 U/ml.. Diabetes Obes. Metab..

[8] Bergenstal R.M., Bailey T.S., Rodbard D. (2017). Comparison of insulin glargine 300 units/mL and 100 units/mL in adults with type 1 diabetes: Continuous glucose monitoring profiles and variability using morning or evening injections.. Diabetes Care.

[9] Haahr H., Heise T. (2014). A review of the pharmacological properties of insulin degludec and their clinical relevance.. Clin. Pharmacokinet..

[10] Heise T., Nosek L., Bøttcher S.G., Hastrup H., Haahr H. (2012). Ultra-long-
acting insulin degludec has a flat and stable glucose-lowering effect
in type 2 diabetes.. Diabetes Obes. Metab..

[11] Alsahli M., Gerich J.E. (2014). Hypoglycemia, chronic kidney disease, and diabetes mellitus.. Mayo Clin. Proc..

[12] Moen M.F., Zhan M., Hsu V.D. (2009). Frequency of hypoglycemia and its significance in chronic kidney disease.. Clin. J. Am. Soc. Nephrol..

[13] Alsahli M., Gerich J. (2015). Hypoglycemia in patients with diabetes and renal disease.. J. Clin. Med..

[14] Inzucchi S.E., Bergenstal R.M., Buse J.B. (2015). Management of hyperglycemia in type 2 diabetes, 2015: a patient-centered approach: update to a position statement of the American Diabetes Association and the European Association for the Study of Diabetes.. Diabetes Care.

[15] (2012). KDOQI Clinical practice guideline for diabetes and CKD: 2012 Update.. Am. J. Kidney Dis..

[16] Pathak R.D., Schroeder E.B., Seaquist E.R. (2016). Severe hypoglycemia requiring medical intervention in a large cohort of adults with diabetes receiving care in U.S. Integrated Health Care Delivery Systems:2005-2011.. Diabetes Care.

[17] Javier Escalada F., Halimi S., Senior P.A. (2018). Glycaemic control and hypoglycaemia benefits with insulin glargine 300 U/mL extend to people with type 2 diabetes and mild-to-moderate renal impairment.. Diabetes Obes. Metab..

[18] Haluzík M., Cheng A., Müller-Wieland D. (2020). Differential glycaemic control with basal insulin glargine 300 U/mL versus degludec 100 U/mL according to kidney function in type 2 diabetes: A subanalysis from the BRIGHT trial.. Diabetes Obes. Metab..

[19] Mauricio D., Gourdy P., Bonadonna R.C. (2021). Glycaemic control with insulin glargine 300 U/ml in individuals with type 2 diabetes and chronic kidney disease: A REALI European Pooled Data Analysis.. Diabetes Ther..

[20] Sullivan S.D., Bailey T.S., Roussel R. (2018). Clinical outcomes in real-world patients with type 2 diabetes switching from first- to second-generation basal insulin analogues: Comparative effectiveness of insulin glargine 300 units/mL and insulin degludec in the DELIVER D+ cohort study.. Diabetes Obes. Metab..

[21] Sullivan S.D., Freemantle N., Menon A.A. https://ada.scientificposters.com/.

[22] Pettus J., Roussel R., Liz Zhou F. (2019). Rates of hypoglycemia predicted in patients with type 2 diabetes on insulin glargine 300 U/ml versus first- and second-generation basal insulin analogs: the real-world lightning study.. Diabetes Ther..

[23] Meneghini L, Cheng A, Evenou P (2020). 1038-P: Insulin glargine 300 U/mL (gla-300) vs. first-generation standard-of-care basal insulin analogs (SOC-BI) in insulin-naïve patients with type 2 diabetes (T2D): impact of renal function on the outcomes of the randomized pragmatic real-life achieve control study. Diabetes.

[24] NCT03703869.

[25] Tirosh A, Khan N, Vargas-Uricoechea H (2021). 743-P: Real-world effectiveness and safety of insulin glargine 300 U/mL (gla-300) in insulin-naïve people with type 2 diabetes (T2DM) and renal impairment (RI): a subgroup analysis of the ATOS study. Diabetes.

[26] Kawaguchi Y., Sawa J., Hamai C., Kumeda Y. (2019). Differential effect of hypoalbuminemia on hypoglycemia on type 2 diabetes patients treated with insulin glargine 300 U/mL and insulin degludec.. Diabetes Ther..

[27] Abdelhafiz A.H., Rodríguez-Mañas L., Morley J.E., Sinclair A.J. (2015). Hypoglycemia in older people - a less well recognized risk factor for frailty.. Aging Dis..

[28] Kirkman M.S., Briscoe V.J., Clark N. (2012). Diabetes in older adults.. Diabetes Care.

[29] Escalada J., Liao L., Pan C., Wang H., Bala M. (2016). Outcomes and healthcare resource utilization associated with medically attended hypoglycemia in older patients with type 2 diabetes initiating basal insulin in a US managed care setting.. Curr. Med. Res. Opin..

[30] Ritzel R., Harris S.B., Baron H. (2018). A randomized controlled trial comparing efficacy and safety of insulin glargine 300 units/ml versus 100 Units/mL in older people with type 2 diabetes: results from the senior study.. Diabetes Care.

[31] Yale J.F., Aroda V.R., Charbonnel B. (2020). Glycaemic control and hypoglycaemia risk with insulin glargine 300 U/mL versus glargine 100 U/mL: A patient-level meta-analysis examining older and younger adults with type 2 diabetes.. Diabetes Metab..

[32] Munshi M.N., Gill J., Chao J., Nikonova E., Patel M. (2018). Insulin glargine 300 u/ml is associated with less weight gain while maintaining glycemic control and low risk of hypoglycemia compared with insulin glargine 100 u/ml in an aging population with type 2 diabetes.. Endocr. Pract..

[33] Charbonnel B., Aroda V.R., Westerbacka J. (2019). #131-LB. Differences in HbA1c reduction between insulin glargine 300 U/mL (Gla-300) and insulin degludec 100 U/mL (IDeg-100) in adults ≥70 years of age with T2DM in the bright trial. ADA 2019.. Diabetes.

[34] Bolli G.B., Cheng A., Charbonnel B. (2021). Glycaemic control and hypoglycaemia risk with insulin glargine 300 U/mL and insulin degludec 100 U/mL in older participants in the BRIGHT trial.. Diabetes Obes. Metab..

[35] Bailey T.S., Wu J., Zhou F.L. (2019). Switching to insulin glargine 300 units/mL in real-world older patients with type 2 diabetes (DELIVER 3).. Diabetes Obes. Metab..

[36] Bonadonna R.C., Mauricio D., Müller-Wieland D. (2021). Impact of age on the effectiveness and safety of insulin glargine 300 U/ml: results from the reali European pooled data analysis.. Diabetes Ther..

[37] Hirose T., Odawara M., Matsuhisa M. (2021). Risk of hypoglycemia in Japanese people with type 2 diabetes mellitus who initiated or switched to insulin glargine 300 U/mL: A subgroup analysis of 12-month post-marketing surveillance study (X-STAR study).. Diabetes Res. Clin. Pract..

[38] Galstyan G.R., Tirosh A., Vargas-Uricoechea H. (2021). 194-OR: Real-world effectiveness and safety of insulin glargine 300 U/mL (Gla-300) in older adults (≥70 Years) with type 2 diabetes (T2DM): A subgroup analysis of the ATOS study.. Diabetes.

[39] Bailey T.S., Pettus J., Roussel R. (2018). Morning administration of 0.4 U/kg/day insulin glargine 300 U/mL provides less fluctuating 24-hour pharmacodynamics and more even pharmacokinetic profiles compared with insulin degludec 100 U/mL in type 1 diabetes.. Diabetes Metab..

[40] Lucidi P., Candeloro P., Cioli P. (2021). Pharmacokinetic and pharmacodynamic
head-to-head comparison of clinical, equivalent doses
of insulin glargine 300 units · mL^−1^ and insulin degludec 100
units · mL^−1^ in type 1 diabetes.. Diabetes Care.

[41] Porcellati F., Lucidi P., Candeloro P. (2019). Pharmacokinetics, pharmacodynamics, and modulation of hepatic glucose production with insulin glargine U300 and glargine U100 at steady state with individualized clinical doses in type 1 diabetes.. Diabetes Care.

[42] Heise T., Kaplan K., Haahr H.L. (2018). Day-to-day and within-day variability in glucose-lowering effect between insulin degludec and insulin glargine (100 U/mL and 300 U/mL): a comparison across studies.. J. Diabetes Sci. Technol..

[43] Home P.D., Bergenstal R.M., Bolli G.B. (2015). New insulin glargine 300 units/ml versus glargine 100 units/ml in people with type 1 diabetes: a randomized, phase 3a, open-label clinical trial (EDITION 4).. Diabetes Care.

[44] Danne T., Matsuhisa M., Sussebach C. (2020). Lower risk of severe
hypoglycaemia with insulin glargine 300 U/ mL *versus* glargine
100 U/ mL in participants with type 1 diabetes: a meta-analysis of
6-month phase 3 clinical trials.. Diabetes Obes. Metab..

[45] Danne T., Tamborlane W.V., Malievsky O.A. (2020). Efficacy and safety of insulin glargine 300 Units/mL (gla-300) versus insulin glargine 100 Units/mL (gla-100) in children and adolescents (6–17 years) with type 1 diabetes: results of the edition junior randomized controlled trial.. Diabetes Care.

[46] Matsuhisa M., Koyama M., Cheng X. (2016). New insulin glargine 300 U/ml versus glargine 100 U/ml in Japanese adults with type 1 diabetes using basal and mealtime insulin: glucose control and hypoglycaemia in a randomized controlled trial (EDITION JP 1).. Diabetes Obes. Metab..

[47] Mathieu C., Weisnagel S.J., Stella P., Bruhwyler J., Alexandre K. (2020). Impact of switching from twice-daily basal insulin to once-daily insulin glargine 300 U/mL in people with type 1 diabetes on basal–bolus insulin: phase 4 optimize study.. Diabetes Ther..

[48] Rea R.R., Dib S.A., de Paula M.A. (2019). P146: Impact of switching from twice-daily (bid) basal insulin to once-daily (OD) insulin glargine 300 U/ml (GLA-300) in patients with type 1 diabetes (T1D)-phase 4 TOP1 trial, Brazilian cohort. 22nd Brazilian Diabetes Society Congress.. Diabetol. Metab. Syndr..

[49] Fritsche A, Pscherer S, Anderten H, Pegelow K, Seufert J, Pfohl M (2018). Switching to insulin glargine 300 U/mL (Gla-300) improves glycemic control after failure of basal-bolus therapy (BBT) with other basal insulins (BI) in patients (Pts) with type 1 diabetes (T1DM). Diabetes.

[50] Pujante A.P., Rodríguez E.R., García U.F. (2019). Experience after switching from insulin glargine U100 to glargine U300 in patients with type 1 diabetes mellitus. A study after one year of treatment in real life.. Endocrinología, Diabetes y Nutrición.

[51] Quansah KA, Sauriol L, Kukaswadia AA, Bremner S, Millson B (2018). Persistence with insulin glargine 300 IU/mL compared with other basal insulins-a canadian retrospective cohort study. Diabetes.

[52] van Mark G., Lanzinger S., Sziegoleit S. (2019). Characteristics of patients with type-1 or type-2 diabetes receiving insulin glargine u300: an analysis of 7268 patients based on the DPV and DIVE Registries.. Adv. Ther..

[53] Pang T., Bain S.C., Black R.N.A. (2019). A multicentre, UK, retrospective, observational study to assess the effectiveness of insulin glargine 300 units/ml in treating people with Type 1 diabetes mellitus in routine clinical practice (SPARTA).. Diabet. Med..

[54] Oriot P., Jérémie W., Buysschaert M. (2018). Outcomes of glycemic control in type 1 diabetic patients switched from basal insulin glargine 100 U/ml to glargine 300 U/ml in real life.. Expert Rev. Endocrinol. Metab..

[55] Abitbol A., Brown R.E., Jiandani D., Sauriol L., Aronson R. (2019). Real-world health outcomes of insulin glargine 300 U/mL vs. insulin glargine 100 U/mL in adults with type 1 and type 2 diabetes in the canadian LMC diabetes patient registry: The REALITY Study.. Can. J. Diabetes.

[56] Laviola L., Porcellati F., Bruttomesso D., Larosa M., Rossi M.C., Nicolucci A. (2021). Comparative effectiveness of switching from first-generation basal insulin to glargine 300 U/ml or degludec 100 U/ml in type 1 diabetes: The restore-1 study.. Diabetes Ther..

[57] Conget I., Mangas M.Á., Morales C. (2021). Effectiveness and safety of insulin glargine 300 U/mL in comparison with insulin degludec 100 u/ml evaluated with continuous glucose monitoring in adults with type 1 diabetes and suboptimal glycemic control in routine clinical practice: the onecare study.. Diabetes Ther..

[58] Matsuhisa M., Koyama M., Cheng X. (2016). Sustained glycaemic control and less nocturnal hypoglycaemia with insulin glargine 300U/mL compared with glargine 100 U/mL in Japanese adults with type 1 diabetes (EDITION JP 1 randomised 12-month trial including 6-month extension).. Diabetes Res. Clin. Pract..

[59] Matsuhisa M., Odawara M., Hirose T. (2021). Real-world data on the use of insulin glargine 300 U/mL in Japanese patients with type 1 diabetes: twelve-month results from a post-marketing surveillance study (X-STAR study).. Expert Opin. Pharmacother..

[60] Home P.D., Bergenstal R.M., Bolli G.B. (2018). Glycaemic control and hypoglycaemia during 12 months of randomized treatment with insulin glargine 300 U/mL versus glargine 100 U/mL in people with type 1 diabetes (EDITION 4).. Diabetes Obes. Metab..

[61] Hassanein M., Buyukbese M.A., Malek R. (2020). Real-world safety and effectiveness of insulin glargine 300 U/mL in participants with type 2 diabetes who fast during Ramadan: The observational ORION study.. Diabetes Res. Clin. Pract..

